# Comparison of postoperative morbidity between conventional cold dissection and bipolar electrocautery tonsillectomy: which technique is better?^☆^

**DOI:** 10.1016/j.bjorl.2018.12.013

**Published:** 2019-03-07

**Authors:** Mohammad Reza Mofatteh, Forod Salehi, Mehran Hosseini, Mahsa Hassanzadeh-Taheri, Gholamreza Sharifzadeh, Mohammadmehdi Hassanzadeh-Taheri

**Affiliations:** a Birjand University of Medical Sciences, Faculty of Medicine, Department of Ears, Nose and Throat, Birjand, Iran; b Birjand University of Medical Sciences, Faculty of Medicine, Department of Cardiology, Birjand, Iran; c Birjand University of Medical Sciences, Cellular and Molecular Research Center, Birjand, Iran; d Birjand University of Medical Sciences, Faculty of Medicine, Birjand, Iran; e Birjand University of Medical Sciences, Faculty of Health, Birjand, Iran; f Birjand University of Medical Sciences, Faculty of Medicine, Department of Anatomy, Birjand, Iran

**Keywords:** Tonsillectomy, Cold dissection, Bipolar electrocautery, Outcome, Tonsilectomia, Dissecção a frio, Eletrocauterização bipolar, Desfecho

## Abstract

**Introduction:**

Tonsillectomy is one of the most common surgeries in the head and neck worldwide. This operation is carried out by different methods, the most frequent of which are the cold dissection and bipolar electrocautery techniques.

**Objective:**

This study was conducted to assess and compare postoperative morbidity between cold dissection and bipolar electrocautery.

**Methods:**

This prospective randomized clinical trial was performed on 534 patients who underwent tonsillectomy in Vali-e-Asr Hospital of Birjand, east of Iran from October, 2013 to October, 2015. The patients were systematically selected for cold dissection technique or bipolar electrocautery technique groups. Time of surgery, amount of intraoperative blood loss, postoperative hemorrhage, the intensity of local pain 4 and 24 hours after operation and nausea and/or vomiting were recorded and compared in the two groups to decide which technique is better. The data were analyzed in SPSS software (ver-22). The *p*-value less than 0.5 was considered significant.

**Results:**

In this study, 51.7% of the cold dissection technique patients and 50.6% of the bipolar electrocautery technique participants were male. Compared to the cold dissection technique, the average intraoperative blood loss was significantly lower (*p* < 0.001) in the bipolar electrocautery technique group, while the intensity of local pain 4 and 24 hours after the operation was significantly higher (*p* < 0.001). Other variables showed no significant differences between the two groups.

**Conclusion:**

Based on the findings of the present investigation, the bipolar electrocautery technique is suggested for tonsillectomy in children, while the cold dissection technique is preferred for adult patients.

## Resumo

**Introdução:**

A tonsilectomia é uma das cirurgias mais comuns de cabeça e pescoço em todo o mundo. Essa cirurgia é feita por diferentes métodos, os mais frequentes são a dissecção a frio e por eletrocauterização bipolar.

**Objetivo:**

Este estudo foi feito para avaliar e comparar a morbidade pós-operatória na dissecção a frio e eletrocauterização bipolar.

**Método:**

Este ensaio clínico prospectivo e randomizado foi feito em 534 pacientes submetidos a tonsilectomia no Vali-e-Asr Hospital de Birjand, no leste do Irã, de outubro de 2013 a outubro de 2015. Os pacientes foram selecionados de forma sistemática para o grupo submetido à técnica de dissecção a frio ou para o grupo com uso da técnica de eletrocauterização bipolar. Para a avaliação acerca da melhor técnica, os seguintes parâmetros foram registrados e comparados entre os dois grupos: tempo de cirurgia, quantidade de perda sanguínea intraoperatória, hemorragia pós-operatória, intensidade da dor local 4 e 24 horas após a cirurgia e ocorrência de náuseas e/ou vômitos. Os dados foram analisados no software SPSS (versão 22). O valor de *p* inferior a 0,5 foi considerado significante.

**Resultados:**

Neste estudo, 51,7% dos participantes do grupo técnica de dissecção a frio e 50,6% do grupo técnica de eletrocauterização bipolar eram do sexo masculino. No grupo operado pela técnica de eletrocauterização bipolar a média de perda sanguínea intraoperatória foi significantemente menor (*p* < 0,001) em comparação à técnica de dissecção a frio, enquanto a intensidade da dor local 4 e 24 horas após a cirurgia foi significativamente maior (*p* < 0,001). As outras variáveis não apresentaram diferenças significantes entre os dois grupos.

**Conclusão:**

Com base nos achados da presente investigação, para a tonsilectomia em crianças sugere-se o uso da técnica de eletrocauterização bipolar, enquanto a técnica de dissecção a frio é recomendada para pacientes adultos.

## Introduction

The palatine tonsils are two lymphatic tissue masses located in the tonsillar fossae on the lateral side of throat or oropharynx.^1^ They are part of the immune system collaborating in the defense of the human body against respiratory infections.^1^ They are prone to become inflamed and enlarged, in a clinical condition called tonsillitis. When these conditions become frequent and severe, or cause complications that affect the patients’ breathing and swallowing, obstructing the upper airway, the physician usually suggests surgical removal of the tonsils.^1^

Tonsillectomy is one of the oldest and most widespread surgical procedures in the field of otorhinolaryngology, and is carried out worldwide. It dates back to 3000 years ago with the first report referring to Hindu medicine about 1000 years B.C.^2^ Cornelius Celsus a Roman surgeon, performed this operation for the first time using his fingernails in 40 AD.^3^, ^4^ He also described scraping the tonsils and cutting them out by a hook-like instrument.^3^ At the beginning of the twentieth century, Worthington (1907) and Waugh (1909) described the technique of tonsillectomy via a dissection method.^5^, ^6^ In 1909, a surgeon named Cohen adopted ligature of bleeding vessels to control perioperative bleeding, and thereafter, tonsillectomy became a common and safe procedure in hospitals around the world.^3^ Remington-Hobbs in 1968^7^ and Haase and Noguera in 1969^8^ introduced the application of diathermy in this procedure. In 1982 Goycolea described electrodissection by using monopolar diathermy^4^, ^9^ and Pang, 10 years later, reported the first tonsillectomy by bipolar electrocautery.^10^

Nowadays, tonsillectomy is performed with a variety of procedures and techniques such as: conventional cold dissection, mono and bipolar electrocauteries, cryosurgery, application of thermal welding system and ultrasonic scalpel, coblation excision, radiofrequency and laser.^11^ Despite the different techniques available for tonsillectomy, there is no consensus and conclusive evidence in the literature to date on the optimum or the best method of performing the tonsillectomy, and none of the techniques has been accepted as the best one universally.^12^, ^13^, ^14^

Each of these techniques has advantages and also disadvantages. These methods have frequently been compared with each other by different investigators around the world, addressing especially the conventional cold dissection technique (CDT) versus the bipolar electrocautery technique (BET). However, findings vary in this regard, possibly due to differences of such parameters as: race, number of cases under study, ecological conditions, lifestyle, the skill of the surgeon, the time of investigation, etc. Hence, we decided to assess and compare the complications of the two different and most commonly used techniques of CDT and BET in admitted patients in Vali-e-Asr Hospital of Birjand, east of Iran, from Oct. 2013 to Oct. 2015.

## Methods

Institutional review and approval from the ethics committee was obtained (no. 01.09.1393). This is a prospective, randomized, double-blind controlled clinical trial (RCT). The patients with obstruction of the upper airway, chronic or recurrent tonsillitis or both were included in the study. They were admitted to the Ear, Nose and Throat (ENT) department of Vali-e-Asr Hospital of Birjand University of Medical Sciences from Oct. 2013 to Oct. 2015 and were scheduled for elective tonsillectomy. The age- matched participants were randomly allocated into BET or CDT tonsillectomy groups at a ratio of 2:1.

Exclusion criteria were as follows: a history of tonsillitis within last two weeks, hemorrhagic diathesis, hematological disorders, congenital malformed tonsils, cranio-facial malformations, asymmetric tonsil appearance, neurological disorders, sensitive to anesthetic drugs, peritonsillar abscess (quinsy), suspected or confirmed tonsillar malignancy and diabetes.

Before an operation, the procedure was explained in detail to the patients or parents. A detailed history was taken, and a general examination of throat, ears, nose and neck was performed. Cell blood count (CBC), erythrocyte sedimentation rate (ESR), hemoglobin (Hb), partial thrombin (PT), partial thrombin time (PTT), platelet count and blood grouping were evaluated, and for patients over 40 years of age, electrocardiography and chest X-ray were requested.

Thereafter, all candidates for tonsillectomy were positioned on the operating table, in Rose's position (supine with a sandbag between the shoulder, neck extension and head support) and anesthetized generally with a standard protocol. The patients’ mouth was held open with Boyle-Davis gag for adequate exposure of the oropharynx. The patients were operated by the CDT or the BET and their tonsils removed. All surgeries were performed by the same surgeon.

Tonsillectomy in the CDT group began by cutting the anterior pillar with a tonsil knife. After identifying the loose connective tissue beneath the tonsil, it was dissected from the superior pole toward the lower pole by a blunt dissector, clamped and removed completely by using Eve's wire snare. Following dissection, the tonsillar fossa was packed with a cotton swab for a few minutes and then, the other tonsil was similarly removed. Finally, the gauzes were removed and when necessary, absorbable sutures were used to secure hemostasis.

In the BET group, Bayonet bipolar forceps electrocautery (Martin, made in Germany) (set power on 40–50 W) was used. In this method, a palatoglossal incision was done from superior pole toward inferior pole using the tip of a bipolar forceps. When the peritonsillar loose connective tissue was identified, the tonsil was completely removed. During dissection, encountered vessels were cauterized and then separated from the tonsil. Any further hemosthasis of the tonsillar fossa was secured by coagulation with the bipolar forceps. After complete hemostasis of both tonsillar fossae was secured, the gauzes were removed. The duration of surgery was monitored (in minutes) by a nurse, registered from placement of Davis mouth gag up to its removal by the surgeon.

In this investigation, the variables include time of surgery, amount of intraoperative blood loss, postoperative hemorrhage, the intensity of local pain 4 and 24 h after operation and presence of nausea and/or vomiting were assessed. These variables were measured by an expert nurse blinded to which patient had which surgical technique.

**Figure 1:**
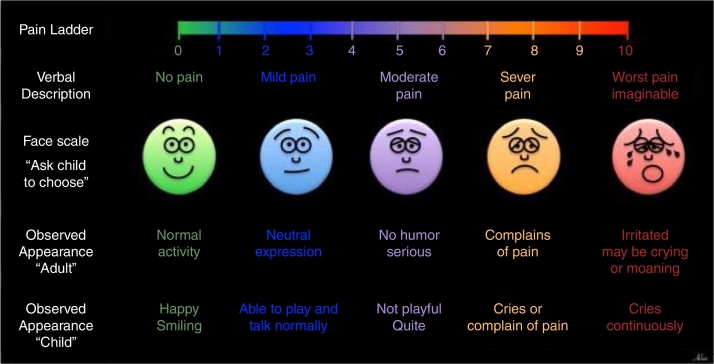
A schematic picture for assess the pain intensity include; Pain Ladder for adult patients and Face scale for children

The pain intensity was estimated using a visual analog scale (VAS) at 4 h and 24 h postoperative. For adult patients, a numerical pain score ladder was used to express pain intensity, whereas, Faces pain scale was employed for pediatric patients (Fig. 1). Accordingly, before surgery, the child and parents were instructed on the use of the pain scales mainly for face scale by an experienced pediatric nurse.^15^

The amount of intra-operative blood loss was measured by calculating weight differences of gauzes before and after use in surgery and adding it to the total weight of blood aspirated in the suction bottle.

The patients were discharged the day after surgery with an oral acetaminophen prescription for pain control every 8 h from the first postoperative day (650 mg for adult and 15 mg/kg for pediatric), if needed. A checklist was given to adult patients or parents of pediatric patients to fill out every day (for next 10 days) that included: postoperative hemorrhage, nausea and/or vomiting and body temperature. Finally, all patients were examined on the 10th postoperative day and their postoperative course evaluated.

The statistical analyses were performed using the SPSS 22 statistical software (IBM, USA). The data were statistically evaluated by the Mann–Whitney *U* and Chi-square tests. All value expressed as the mean ± standard deviation. Values were considered significant at *p* < 0.05.

## Results

This study was carried out on 534 patients who were scheduled for elective tonsillectomy by the cold dissection or the bipolar electrocautery methods.

A total of 178 patients were operated with the CDT and 356 patients with the BET. The mean age of the patients were 11.7 ± 8.8 and 11.87 ± 8.8 years in the CDT and the BET groups, respectively (*p* = 0.59). Furthermore, 92 patients (51.7%) in the CDT group and 180 patients (50.6%) in the BET group were males (*p* = 0.81). No significant differences were found in these demographic characteristics between the patients of the two groups (Table 1). The main indications for surgery were recurrent tonsillitis (34.3%), upper airway obstruction (27.5%), both concurrently (34.5%) and other (3.7%).

**Table 1:** Demographic information of studied patients operated with different two techniques (cold dissection and bipolar electrocautery).

	All patients	CDT group	BET group	*p*-value
Number of patients	534	178	356	
Age range (years) (mean)	3–58	11.74 ± 8.3	11.87 ± 8.8	0.59
Gender				0.81
Male	272	92 (51.7%)	180 (50.6%)	
Female	256	86 (48.3%)	170 (49.4%)	

While the mean duration time of operation was greater in the BET group (12 ± 4.7 min) than the CDT group (11.14 ± 3 min), the difference was not statically significant (*p* = 0.32).

The mean amount of intra-operative blood loss was significantly higher in the CDT group when compared with that of the BET group (*p* < 0.001) (Table 2).

**Table 2:** Comparison of the means ± SD of studied variables in the two groups operated with cold dissection (CDT) and bipolar electrocautery (BET).

Studied groups variables	BET group (*n* = 356) SD ± X	CDT group (*n* = 178) SD ± X	Mann-Whitney (*p* - value)
Duration of surgery (min)	12 ± 4.74	11.14 ± 3	*p* = 0.32

*Intra-operative blood loss(gram)*			
<12 years old	35.48 ± 11.15	43.84 ± 8.48	*p* < 0.001
>12 years old	38.12 ± 11.92	48.10 ± 11.65	*p* < 0.001
Total	36.26 ± 11.43	42.2 ± 9.8	*p* < 0.001

*4* *h postoperative pain score*			
<12 years old	1.81 ± 0.92	1.52 ± 0.55	*p* < 0.001
>12 years old	2.23 ± 1.09	1.74 ± 0.59	*p* = 0.002
Total	1.93 ± 0.99	1.59 ± 0.57	*p* < 0.001

*24* *h postoperative pain*			
<12 years old	2.53 ± 0.91	2.53 ± 0.91	*p* < 0.001
>12 years old	3.15 ± 1.06	1.86 ± 0.83	*p* < 0.001
Total	2.72 ± 1	1.93 ± 0.78	*p* < 0.001

*Nausea and/or vomiting*			
Yes	64 (18)	25 (14)	*X*^2^ = 1.32
No	292 (82)	153 (86)	*p* = 0.25
Postoperative hemorrhage	1 (0.28%)	1 (0.56%)	–

The intensity of local pain 4 and 24 h after operation increased significantly in both time points in the BET group than the CDT group (*p* < 0.001) (Table 2).

Nausea and/or vomiting showed no significant differences in comparison between the two groups.

## Discussion

The postoperative morbidity of tonsillectomy remains high, and several techniques have evolved over the years to decrease these occurrences (mainly blood loss and postoperative pain).^16^ Complications of tonsillectomy by cold dissection^17^ and also bipolar electrocautery methods^18^ have already been studied by our research team. In the present study, morbidity rates from these two techniques were assessed as well as compared.

While there is controversy on which technique is the best for tonsillectomy, it is well accepted that the ideal technique should be quick, decrease postoperative pain, lead to minimal perioperative bleeding and also demonstrate maximal safety and effectiveness.^15^, ^19^

According to the findings of this research the intraoperative blood loss was significantly less in the BET group than the CDT group, which is in good agreement with other similar studies and supports the results of previously published articles by Hashemi et al. (2002),^20^ Silveria et al. (2003),^15^ Kousha et al. (2007),^21^ Adoga (2011),^19^ Ahmad & Bassiouny (2009),^22^ Guragain et al. (2010),^14^ Vithyathil et al. (2017)^13^ and Ali et al. (2014).^12^ Blood loss is a very important parameter, particularly in childrens’ surgery, because they have a small amount of circulating blood volume where bleeding may cause disorders such as mental and physical fatigue and lead to unfavorable results.^23^, ^24^ Since in bipolar electrocautery procedure dissection of tonsil and coagulation of vessels are performed simultaneously, it can be done with no or little bleeding, which is a major advantageous of this method especially in children.^25^, ^26^

Postoperative hemorrhage is the most potential life-threatening complication that occurs in less than 10% of the patients, and most often in the first 24 h of operation.^15^ Windfuhr and Seehafer (2001) based on a study was performed on 602 patients, divided postoperative bleeding into two categories, primary hemorrhage, that occurs during the first 24 h of operation (<24 h) and secondary hemorrhage, that happens after 24 h (>24 h).^27^ The frequency of this complication depends on the procedure employed and varies from 1% to 6% in different patients.^11^ In previous studies, the rate of bleeding after tonsillectomy has been reported 1.3% for CDT and 3.9% for BET groups.^16^ The rates for secondary hemorrhage have been 2.3% for BET and 1% with CDT.^28^ Lowe and colleagues (2007) concluded that diathermy tonsillectomy is associated with high risk of secondary bleeding.^29^ This parameter occurred rarely in our study (one case for each group) and their rates were 0.56% and 0.28% for the CDT and the BET groups respectively. In consonance with our study, percentage of postoperative hemorrhage has been low and statically insignificant in some studies.^30^ Silveria et al. (2003) reported one case in each group of CDT and BET groups.^11^ In Adoga's study there was no postoperative bleeding,^19^ in Yilmaz et al.’s investigation (2012) only one case in CDT had primary postoperative hemorrhage,^31^ in Ali et al.’s research (2014) the incidence of this parameter was 5.6% in BET group^12^ and in Chettri's study 3.06% in CDT group.^23^, ^24^

One of the other parameters in the present investigation was duration of surgery. Reduced surgery time means less time under anesthesia and in turn faster recovery. The operation time in our study, as opposed to most previous investigation, was longer in the BET group than the CDT group, though not significantly. Similarly, in some other studies by Kujawski et al. (1997),^32^ Lassaletta et al. (1997),^33^ Bukhari and Al-Ammar (2007)^34^ and Yilmaz et al. (2012)^31^ the operation time were not significantly different. Nonetheless, in contrast to our study and the above-mentioned studies, the majority of research carried out by Silveria et al. (2003),^11^ Kousha et al. (2007),^21^ Guragain et al. (2010),^14^ Adoga (2013),^19^ Ali et al. (2014)^12^ and Vithayathil et al. (2017)^13^ report a significantly longer duration of surgery in the CDT group. Prolongation of the BET surgery in our study can be attributable to factors including the surgeon's skill and speed of operation in the cold dissection procedure. In this regard, our surgeon shows the shortest operation time in the cold dissection method as compared with similar studies. In the present study, the mean time of operation in the CDT group was 11.14 ± 3 min, while this time in other studies have been as follow: in Ali et al. = 13.5′,^12^ Kusha et al. = 15′,^21^ Chettri et al. = 16.5′,^24^ Vithayathil et al. = 18′,^13^ Silveria et al. = 21′,^11^ Guragain et al. = 23′,^14^ Bukhari & Al-Ammar = 23′^34^ and Yilmaz et al. = 27′.^31^

Nausea and/or vomiting are another complication covered in this study. Although the incidence of this variable was greater in the BET group than the CDT, no significant difference was found between the two groups. This result is a good match with the finding of Stavroulaki et al. (2007)^16^ who compared morbidity of tonsillectomy between cold dissection and thermal cautery methods.^16^ Severe vomiting may cause dehydration whereby patients require hospitalization and intravenous fluid intake.^16^ Fortunately, in this study the involved patients had mild vomiting and did not need hospitalization.

Pain intensity was a furtherer parameter in this study, which was recorded and compared between the BET and the CDT groups on two occasions, 4 and 24 h after operation. In both of studied time points, the patients undergoing tonsillectomy with the BET experienced higher pain intensity than the CDT group. Similarly, higher pain intensity scores with BET have been reported by Gendy et al. (2005)^28^ and Silveria et al. (2003).^11^ In Ali et al.’s study (2014),^12^ although, the initial postoperative pain was not different statistically between BET and CDT groups, later, on 7th and 14th days after surgery, the severity of pain was significantly higher in patients who underwent tonsillectomy with BET.^12^ In studies carried out by Chettri et al. (2010),^23^, ^24^ Adoga (2011)^19^ and Bukhari and Al-Ammar (2007)^34^ a significantly greater percentage of patients in the BET group complained of higher pain intensity than the CDT group, which is consistent with our findings. Fida and Sendi (2013),^15^ Yilmaz et al. (2012)^31^ and Stavroulaki et al. (2007)^16^ assessed and compared pain intensity between two techniques of tonsillectomy cold dissection and thermal welding. Their findings showed that the severity of pain was lower in CDT group when compared with BET group, which is inconsistent with our results. In Alam et al. (2011),^35^ Vithayathil and colleagues (2017)^13^ and Hashemi et al. (2002)^20^ pain intensity showed no significant difference between two studied groups BET and CDT. The observed differences of pain intensity in different studies could possibly be related to various factors such as length of disease, amount and severity of employed energy during surgery, the generated heat leading to tissue burning, size of the cut area, ability to tolerate pain etc.

## Conclusion

Based on the findings of the present study, intra-operative blood loss was reduced significantly in the BET group. This parameter is an important advantage for tonsillectomy especially in pediatric patients as they have a low blood volume. Hemorrhage in these patients may lead to unfavorable disorders. Hence, the BET is suggested for tonsillectomy in children. The other parameters assessed in this study were more desirable in the CDT group; therefore, this technique is preferred for tonsillectomy in adult patients.

## Funding

This work was financially supported by Birjand Universityof Medical Sciences (Grant no. 1012).

## Conflicts of interest

The authors declare no conflicts of interest.
